# OEDL: an optimized ensemble deep learning method for the prediction of acute ischemic stroke prognoses using union features

**DOI:** 10.3389/fneur.2023.1158555

**Published:** 2023-06-21

**Authors:** Wei Ye, Xicheng Chen, Pengpeng Li, Yongjun Tao, Zhenyan Wang, Chengcheng Gao, Jian Cheng, Fang Li, Dali Yi, Zeliang Wei, Dong Yi, Yazhou Wu

**Affiliations:** ^1^Department of Health Statistics, College of Preventive Medicine, Army Medical University, Chongqing, China; ^2^Department of Neurology, Taizhou Municipal Hospital, Taizhou, Zhejiang, China; ^3^Department of Radiology, Taizhou Municipal Hospital, Taizhou, Zhejiang, China; ^4^Department of Health Education, College of Preventive Medicine, Army Medical University, Chongqing, China

**Keywords:** MRI, radiomics, deep learning, ensemble learning, metaheuristic algorithms, ischemic stroke

## Abstract

**Background:**

Early stroke prognosis assessments are critical for decision-making regarding therapeutic intervention. We introduced the concepts of data combination, method integration, and algorithm parallelization, aiming to build an integrated deep learning model based on a combination of clinical and radiomics features and analyze its application value in prognosis prediction.

**Methods:**

The research steps in this study include data source and feature extraction, data processing and feature fusion, model building and optimization, model training, and so on. Using data from 441 stroke patients, clinical and radiomics features were extracted, and feature selection was performed. Clinical, radiomics, and combined features were included to construct predictive models. We applied the concept of deep integration to the joint analysis of multiple deep learning methods, used a metaheuristic algorithm to improve the parameter search efficiency, and finally, developed an acute ischemic stroke (AIS) prognosis prediction method, namely, the optimized ensemble of deep learning (OEDL) method.

**Results:**

Among the clinical features, 17 features passed the correlation check. Among the radiomics features, 19 features were selected. In the comparison of the prediction performance of each method, the OEDL method based on the concept of ensemble optimization had the best classification performance. In the comparison to the predictive performance of each feature, the inclusion of the combined features resulted in better classification performance than that of the clinical and radiomics features. In the comparison to the prediction performance of each balanced method, SMOTEENN, which is based on a hybrid sampling method, achieved the best classification performance than that of the unbalanced, oversampled, and undersampled methods. The OEDL method with combined features and mixed sampling achieved the best classification performance, with 97.89, 95.74, 94.75, 94.03, and 94.35% for Macro-AUC, ACC, Macro-R, Macro-P, and Macro-F1, respectively, and achieved advanced performance in comparison with that of methods in previous studies.

**Conclusion:**

The OEDL approach proposed herein could effectively achieve improved stroke prognosis prediction performance, the effect of using combined data modeling was significantly better than that of single clinical or radiomics feature models, and the proposed method had a better intervention guidance value. Our approach is beneficial for optimizing the early clinical intervention process and providing the necessary clinical decision support for personalized treatment.

## Introduction

In recent years, with the increasingly serious aging phenomenon globally, the incidence of major chronic diseases represented by ischemic strokes has also increased (1). Stroke is still the second leading cause of death in the world and the number one cause of acquired long-term disability, especially in China, which is the greatest challenge of stroke in the world, ranking third among the leading causes of death in China, second only to malignant tumors and heart disease (2). Acute ischemic stroke (AIS) is associated with high morbidity, high mortality, and poor prognoses. They have become a major public health problem that cannot be ignored and have brought a great burden to the economy and society. In the context of limited medical resources, it is necessary to prioritize the implementation of nursing care for patients with poor prognoses, thereby reducing the incidence of disability (3). In the era of precise diagnosis and individualized treatment, prognostic classification has become an important strategy for stroke management (4, 5). The early prediction of prognoses is of great significance for improving the efficiency of stroke disease diagnosis and treatment and improving the levels of disease prevention and control (6).

In the past, prognosis evaluations in clinical practice mostly relied on the manual judgments of physicians, which required high-end medical technology and much physician experience, and the prediction effect of this approach was unstable, which limited its clinical promotion (7, 8). As a new non-invasive technique, radiomics can extract high-throughput quantitative information from traditional medical images, enabling the assessment of internal tumor textures that cannot be captured by visual assessments (9, 10). Radiomics aims to extract quantitative and high-dimensional data from digital biomedical images to facilitate the comprehensive exploration of disease information and progression, and it has been widely used in a variety of clinical fields (11, 12). However, previous studies of this kind were mostly limited to radiomics alone and failed to comprehensively predict disease prognoses with clinical and radiomics features (13–17). At present, there is still a lack of relevant research focusing on the predictive value of combined features for stroke prognosis, which has broad research prospects.

Compared with traditional prediction models, deep learning-based prediction models, represented by deep neural networks (DNNs), long short-term memory recurrent neural networks (LSTM-RNNs), and deep belief networks (DBNs), can automate and accurately analyze a large number of features and are suitable for various medical fields (18–21). Ensemble learning has the advantages of fast operation and high accuracy and has been widely used in numerous fields, such as medical treatment, healthcare, and information technology (22). Single machine learning and deep learning have the problems of limited convergence effect and difficulty in optimizing hyperparameters, which affects the improvement of prediction efficiency. Deep ensemble learning is expected to solve these problems and improve the accuracy of the model. Compared with shallow learning models and individual learning models, ensemble deep learning models can perform better on multiple learning tasks. They can also extract deeper essential features during the learning process, which can effectively improve the accuracy of the model prediction results (23).

In previous radiomics studies, the applications of deep ensemble models were relatively lacking (24–26). If the selected network structure and parameter settings are not appropriate, this may increase the complexity of the model and reduce its overall operating efficiency (27). Hence, the parameter optimization and layer number setting steps of deep ensemble models are still key issues that need to be solved (28). To improve the optimization accuracy of these models and reduce the time required for the optimization process, such research usually requires the use of metaheuristic algorithms as optimization strategies (29, 30). However, traditional algorithms often have problems such as slow convergence speeds and ease of falling into local optima (31, 32). Research on optimization algorithms with novel optimization mechanisms, accurate solution methods, and robust computing power is still an important direction for feature and parameter selection.

This study aims to build a stroke prognosis prediction model in a deeply integrated way to provide a reference for the diagnosis and prevention of stroke. We adopt an ensemble concept involving data, methods, and algorithms and achieve excellent classification performance. Our contributions and the innovations of this study can be summarized as follows.

(1) In terms of data fusion, we innovatively extract, select, and fuse clinical features and imaging features. The combined data are beneficial to fully extract information and provide early warning for the prognosis of stroke more comprehensively and effectively.

(2) In terms of the categorical outcome, multicategorical outcome variables (normal group, mild group, and moderate-severe group) are used in this study. Compared with that of two-classification approaches, the multi-classification method is conducive to improving the pertinence of the classification, which is conducive to accurate prognosis judgment and intervention guidance.

(3) In terms of model construction, we innovatively construct the optimized ensemble of deep learning (OEDL) method. We comprehensively selected and integrated multiple deep learning methods to maximize the advantages of each method and verified the performance of the model for classification prediction. Our proposed model increases the diversity of prognosis prediction methods, enriches the methodological content of deep ensemble learning, provides new methods and ideas in its research field and clinical decision support for personalized intervention.

(4) For model optimization, we design a new Big Bang optimization algorithm (BBOA), which aims to implement the optimization process efficiently and accurately and then improve the efficiency of the feature selection and parameter search processes.

## Materials and methods

**Figure 1:**
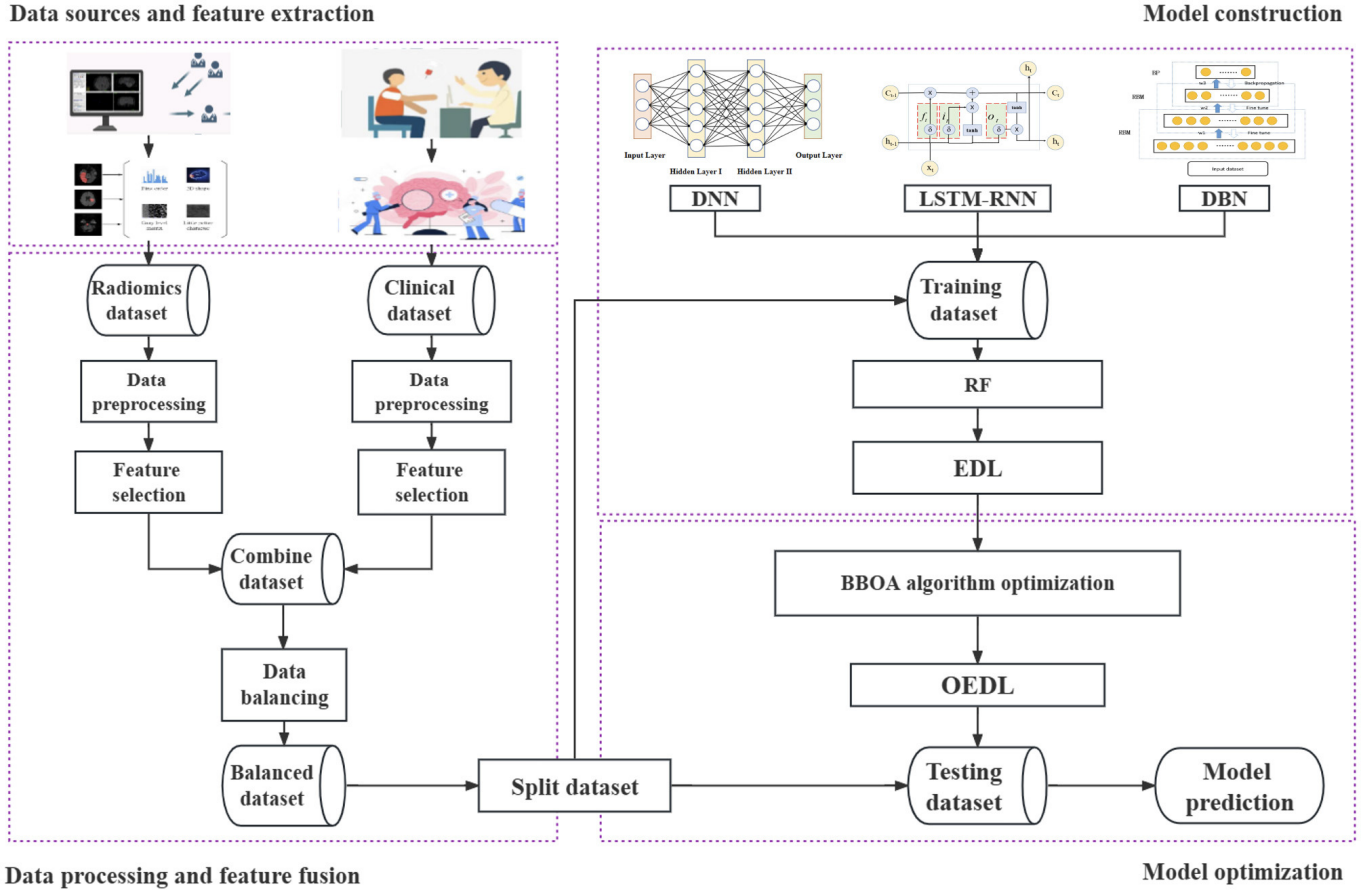
Whole pipeline of the proposed method. The data source and feature extraction, data processing and feature fusion, model construction, model optimization, and other processes are included. Each step is represented by a dotted box.

The data that support the findings of this study are available from the corresponding author upon reasonable request. This study includes the following steps. (1) Data source and feature extraction: The clinical features and radiomics features are extracted in turn. (2) Data processing and feature fusion: The data filling, data noise reduction, data standardization, data screening, data splicing, data balancing, and related steps are performed. (3) Model construction: Clinical features, imaging features, and combined features from the data are included in turn. In this method, the concept of deep integration is used for modeling, and the base learner and the integration mode are selected in turn. (4) Model optimization: The proposed improved metaheuristic algorithm is used to improve the efficiency of the parameter search. Our technical route is shown in Figure 1.

### Data source and feature extraction

This retrospective study was approved by the ethics committee of the Taizhou Municipal Hospital, and the requirement to obtain informed consent was waived. A total of 477 acute ischemic stroke (AIS) patients admitted to the Department of Neurology, Taizhou Municipal Hospital, Zhejiang Province, from January 2020 to April 2021 were recruited. The inclusion criteria were as follows: those who met the AIS diagnostic criteria, had complete clinical data, age more than 18 years. The patient had the first episode, and the MRI images were clear and without artifacts. Severe liver and kidney dysfunction, blood system diseases, malignant tumors, immune system diseases and other diseases, other serious nervous system diseases, and failure to cooperate with clinical treatment or follow-up as required by law were exclusion criteria. A total of 441 cases were eventually included. To ensure that the sample size met the needs of deep learning, we implemented balancing processing for the study data.

The prognosis groupings were based on the National Institute of Health Stroke Scale (NIHSS) at the time of discharge and could be divided into three groups (33, 34): a normal group (<1 point) with 106 cases; a mild group (1–4 points) with 289 cases; and a moderate-severe group (≥ 5 points) with 46 cases. In the following text, we refer to the normal, mild, and model severe groups as groups A, B, and C, respectively. The NIHSS scores could reflect the degrees of neurological deficit in patients and were used as prognostic indicators in this study.

The clinical data included NIHSS score at admission, disease type, OCSP classification, sex, age, body mass index (BMI), systolic blood pressure (SBP), left ventricular hypertrophy (LVH), homocysteinemia, history of hypertension, history of diabetes, history of coronary heart disease (CHD), history of atrial fibrillation, history of drinking, history of smoking, serum total cholesterol (TC), and low-density lipoprotein (LDL). The distribution of baseline data of each group is shown in Section Results of clinical feature selection of the results. Because of the first onset, relevant characteristics such as “stroke history” were not included in this article.

The image data were obtained from cranial MR images, and a Philips Achieve 1.5T scanner was used to obtain these data. The axial DWI sequence was acquired from all patients. To obtain DW images, the following parameters were used: the echo time was 101 ms, the repetition time was 3,211 ms, the number of excitations was 1, the slice thickness was 5 mm, the slice spacing was 1 mm, the acquisition matrix was 230 × 230, and the field of vision was 23 cm ^*^ 23 cm.

**Figure 2:**
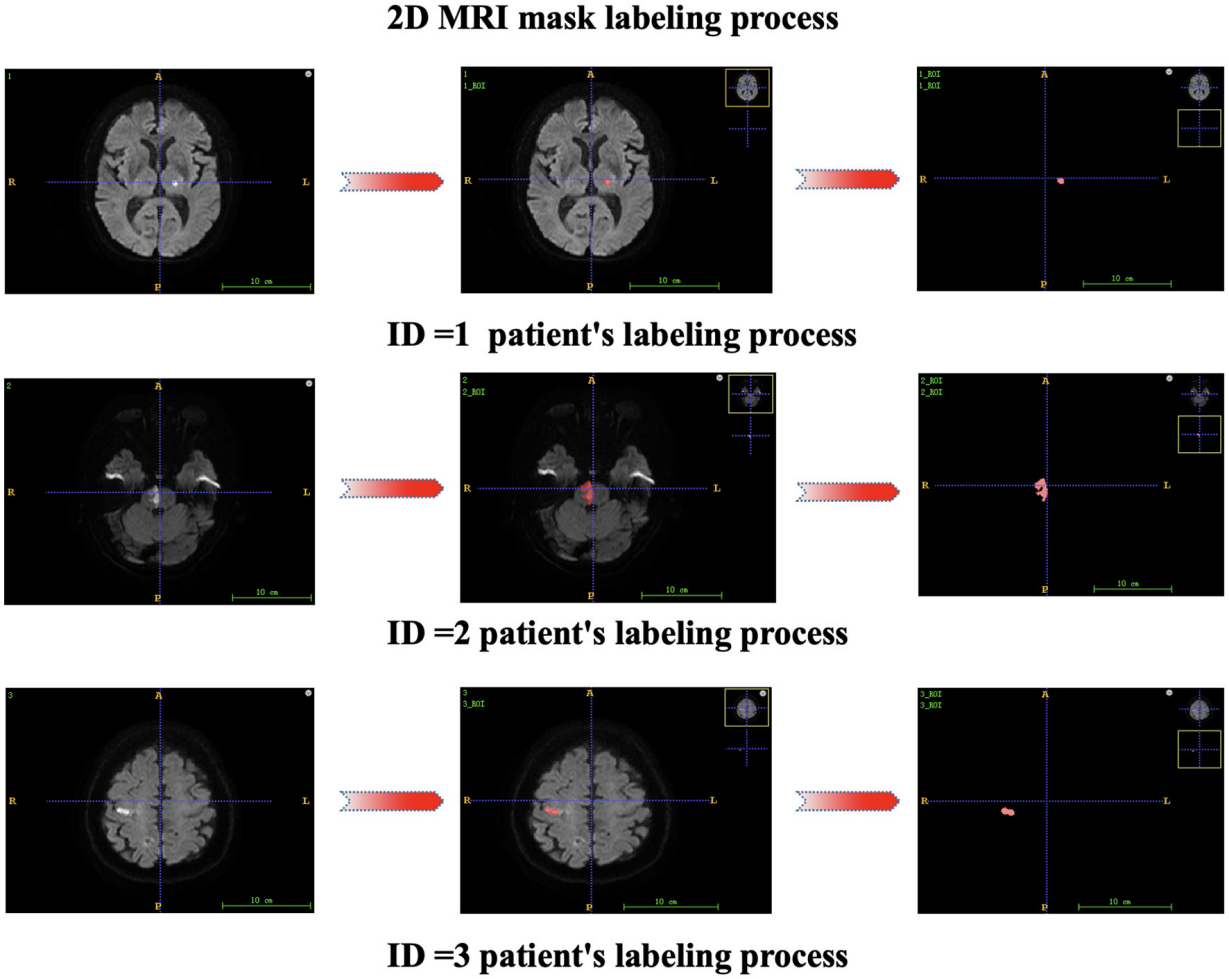
2D mask labeling process for patients.

Each patient's first MR image was collected after admission. Two attending physicians independently segmented the regions of interest (ROIs) from the lesions, and ITK-SNAP 3.6.0 software was used for segmentation to obtain the 3D structural data of the lesions. The radiomics features of each annotated lesion were then obtained by using a radiomics analysis tool (the Pyradiomics package). The 2D mask labeling process for each patient is shown in Figure 2. The radiomics features included shape features (14 features), first-order statistics (162 features), gray-level dependence matrix features (GLDM features, 126 features), gray-level cooccurrence matrix features (GLCM features, 216 features), gray-level run length matrix features (GLRLM features, 144 features), gray-level size zone matrix features (GLSZM features, 144 features), and neighboring gray-tone difference matrix features (NGTDM features, 45 features). Finally, 17 clinical features and 851 radiomics features were initially included in this study.

### Data processing and feature fusion

**Figure 3:**
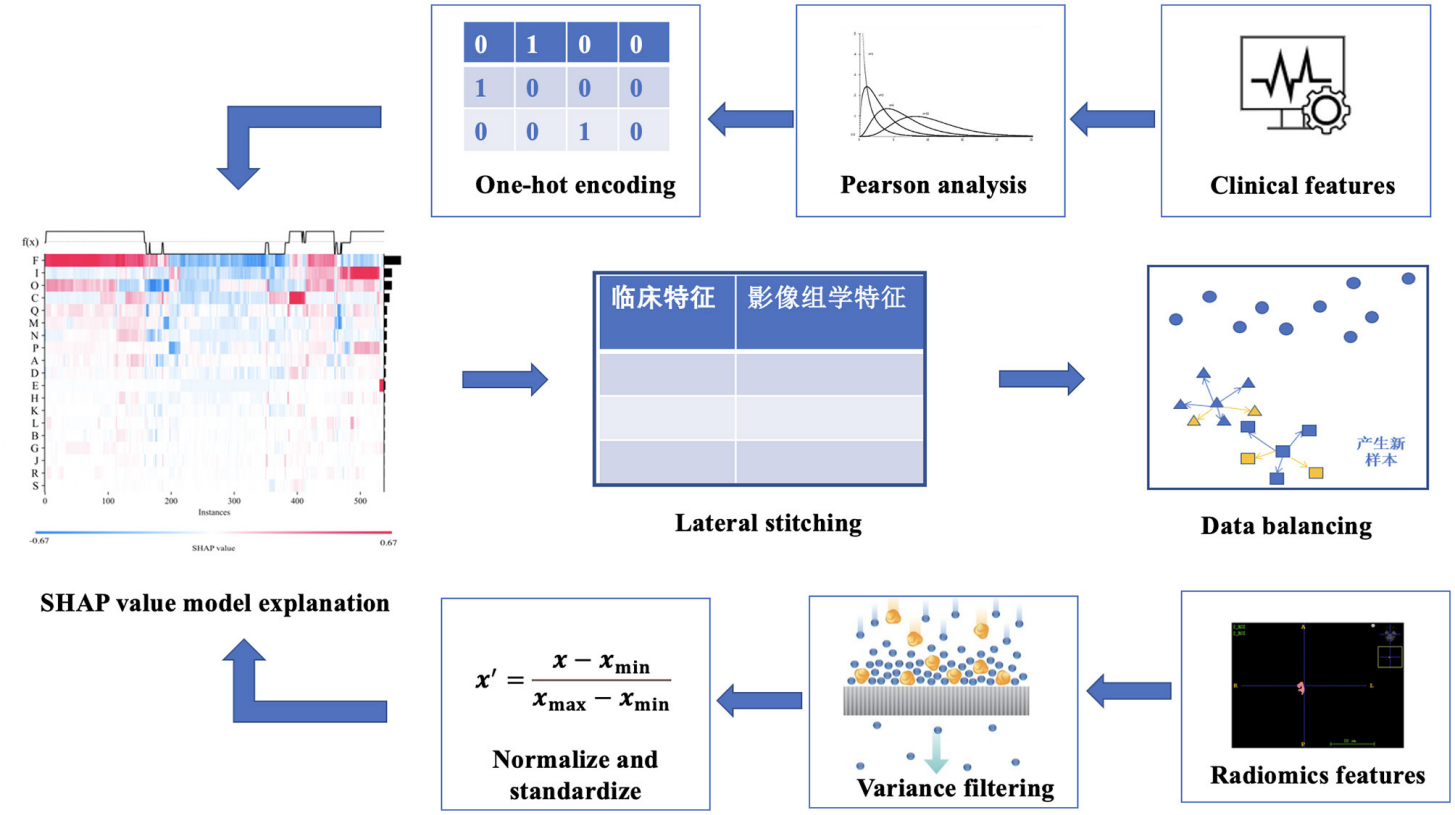
Feature fusion process.

After feature extraction, data preprocessing was performed, including data filling, data noise reduction, data standardization, data screening, data splicing, data balancing, and other steps, as shown in Figure 3.

First, in data imputation, we used multiple imputation methods (35). Multiple imputation is a commonly used method to deal with missing values in data. Its basic principle is to generate multiple complete data sets through simulation, and each data set uses different methods to impute missing values. In this study, we use a multiple imputation method based on the multiple Monte Carlo Method to imputation the samples to reduce the impact of missing data on model construction. The multiple Monte Carlo method is a statistical method that uses multiple independent random samples to estimate the expected value. Suppose the expected value E[*f*(*x*)] of some function *f*(*x*), where *x*∈*R*^*d*^ is a vector of dimension *d*. The formula for the multiple Monte Carlo method is as follows:


(1) $$\begin{array}{l} {\bar{f}}_{n}=\frac{1}{N}\overset{N}{\underset{i=1}{\sum }}f\left(x_{n},i\right) \end{array}$$

(2) $$\begin{array}{l} {\bar{f}}_{m}=\frac{1}{M}\overset{M}{\underset{i=1}{\sum }}{f_{n}}_{\left(j\right)} \end{array}$$

where ${\bar{f}}_{n}$ is the sample mean value of *f*(*x*) obtained from the *n*-th sampling, *x*_*n*_, *i* is the *i*-th sample point obtained from the *n*-th sampling, and *N* is the number of samples for each sampling; ${\bar{f}}_{m}$ is the multiple Monte Carlo estimator obtained by averaging ${\bar{f}}_{n}$ obtained from *n* samples for *m* times, where *n*(*j*) is the sample set used for the *j*-th sample.

Second, for high-dimensional imaging features, a large number of useless noise features will affect the screening of meaningful features and increase the difficulty of model construction (36). In data denoising, we chose the variance selection filtering method to perform variance-based feature screening (37) and then filtered out features with small differences. The variance of each feature was calculated, and features with variances greater than the threshold were selected. If the variance is small, it means that there is a small difference between these samples with respect to the feature, and this feature is not conducive to sample discrimination. We filtered features with zero or less variance to preferentially exclude features with lower contributions.

Third, the data were standardized by distinguishing clinical features from radiologic features. (A) Clinical characteristics were assigned to a range between 0 and 1 by one-hot encoding because one-hot encoding can extend the values of discrete features to a Euclidean geometry space and thus fuse standardized imaging features (38, 39). The mathematical formula for one-hot encoding is as follows. Let the value of a discrete feature *x* with *n* different values be {*x*_1_, *x*_2_, ..., *x*_*n*_}; then, the one-hot encoding of this feature is an *n*-dimensional vector *v*, where if and only if the value of *x* is *x*_*i*_, *v*[*i*] = 1; if *x* is not *x*_*i*_, *v*[*i*] = 0. (B) For radiomics features, we selected a normalization method for feature processing, aiming to eliminate dimensional differences between different features, avoid possible deviation in the model training, improve the convenience of data processing, and speed up the model convergence (40, 41). Normalization helps to ensure dimensional unity between different features, thus improving the robustness and generalizability of the model (42, 43). Normalization refers to scaling the data so that it falls within a specific interval. Standardization (Sz) is the transformation of data into a normal distribution with a mean of 0 and a standard deviation of 1 (44). Suppose that there are *N* samples, each sample has *n* features, and the value of the *i-*th feature of all *N* samples is *x*_*i*1_, *x*_*i*2_, ..., *x*_*in*_. Then, the standardized mathematical formula of the feature is as follows:


(3) $$\begin{array}{l} Sz=\frac{x_{ij}-\mu_{i}}{\sigma_{i}} \end{array}$$

where *μ*_*i*_ denotes the mean of the *i-*th feature over all *N* samples and *σ*_*i*_ denotes the standard deviation of the *i-*th feature over all *N* samples. For the *i-*th feature in each sample, a new value can be obtained from this formula, representing the relative size and distribution of the feature across the entire data set.

Fourth, we use the embedded method to filter and reduce the dimensions of the data. The LightGBM and XGBoost algorithms are selected to perform feature importance scoring and selection, the top 50 most important features in terms of weight are screened out, and the features appearing in both methods and the top 10 features in terms of weight in each method are sorted out. (A) LightGBM is a gradient-boosting framework based on a decision tree (DT). It uses a node segmentation strategy based on leaves, seeks the leaf with the largest gain among all the current leaves, and finally generates a boosted tree (45, 46). The LightGBM algorithm is based on the selection of partition points based on the histogram algorithm and reduces the number of samples and features required in the training and learning processes through two methods, namely, gradient-based one-side sampling (GOSS) and exclusive feature bundling (EFB), to maintain high learning performance and reduce the resource occupation in terms of time and space in the training process (47, 48). Let X^s^ be the input space, *s* be the feature dimension, and *Y* be the output space. The given training dataset is {(*x*_1_, *y*_1_), (*x*_2_, *y*_2_), ..., (*x*_*n*_, *y*_*n*_)}, where ${\vec{x}}_{i}=\left({{\vec{x}}_{i}}^{\left(1\right)},{{\vec{x}}_{i}}^{\left(2\right)},...,{{\vec{x}}_{i}}^{\left(s\right)}\right),i=1,2,...,n$ represents the input instance and {*g*_1_, *g*_2_, ..., *g*_*n*_} represents the negative gradient direction of the loss function relative to the model output at each enhancement iteration. Let *n* represent the number of samples, and let *O* be the training set of the DT on a node. Then, the information gain *V*_*j*|*o*_(*d*) (49) of feature *j* at node *d* can be defined as


(4) $$V_{j|o}(d)=\frac{1}{n_{0}}(\frac{(\sum_{x_{i}\in o:x_{ij}\leq dg_{i}}{)}^{2}\;}{n_{l|o}^{j}(d)}+\frac{(\sum_{x_{i}\in o:x_{ij}>dg_{i}}{)}^{2}\;}{n_{r|o}^{j}(d)})$$

where *n*_0_ = ∑*I*(*x*_*i*_ ∈ *o*), $n_{l|o}^{j}=\sum I[xi\in o]:xij\leq d]$, and $n_{r|o}^{j}=\sum I\left[x_{i}\in o:x_{ij}>d\right]$. (B) The XGBoost algorithm can generate a second-order Taylor expansion of the utilized loss function and obtain the optimal solution for the regular term outside the loss function (50, 51). The larger the weight of a feature and the more times it is selected by the boosted tree, the more important the feature is considered to be (52, 53). Suppose that the model has *t* DTs, *n* represents the total number of samples, *f*_*t*_ represents the *t*-th regression tree, *F* represents the collective space of all DTs, and ${ŷ}_{i}^{{\;}_{t}}$ represents the total predicted value for the *i*-th sample after adding the outputs of the *t* DTs. Then, the predicted value of XGBoost (54) can be expressed as


(5) $${\hat{y}}_{i}^{\left(t\right)}=\sum_{k=1}^{t}f_{k}(x_{i})={\hat{y}}_{i}^{\left(t-1\right)}+f_{t}(x_{i}),f_{k}\in F,i\in n$$

Its loss function is


(6) $$L^{\left(t\right)}=\sum_{i=1}^{n}l(y_{i},{\hat{y}}_{i}^{\left(t\right)})+\sum_{k=1}^{t}\Omega (f_{k})$$

(7) $$\begin{array}{l} \Omega \left(f_{k}\right)=\lambda T+\frac{1}{2}\lambda {\left||w|\right|}^{2} \end{array}$$

where *l* represents the error between the predicted value and the actual value, *T* and *w* represent the number and weight of the leaf nodes, respectively, and γ and λ represent regularization coefficients. The k-th tree is represented by *k*, and the complexity of k trees is represented by $\sum_{k=1}^{t}\Omega (f_{k})$. (C) The Pearson correlation coefficient can measure the strength and direction of the linear relationship between two variables, and different correlation coefficients can be selected according to different data characteristics (55). If two features have a high correlation, this indicates that the information contained in the two features is highly similar, and too much similar information can reduce the performance of the chosen algorithm (56). Hence, only one feature must be reserved for features whose correlations are higher than a certain threshold. To avoid the negative impact of collinearity features on outcome variables, we randomly retained only one of many features with Pearson correlation coefficients greater than the threshold (0.9 in our study). (D) In this study, the SHAP model interpreter tool is used to explain the operation mechanism of the model. SHAP can construct a weighted explanatory model to calculate the contribution of each feature to the results (57, 58). In the interpretation of radiomics and clinical features using LightGBM and XGBoost, respectively, each sample can generate a predictive value, and the SHAP value is expressed as *f* (*x*), which can represent the numerical value assigned to each feature in a sample. Red represents features that act positively, and blue represents features that act negatively (5). After the screening of clinical and radiomics features, the combined features were constructed by stitching.

**Figure 4:**
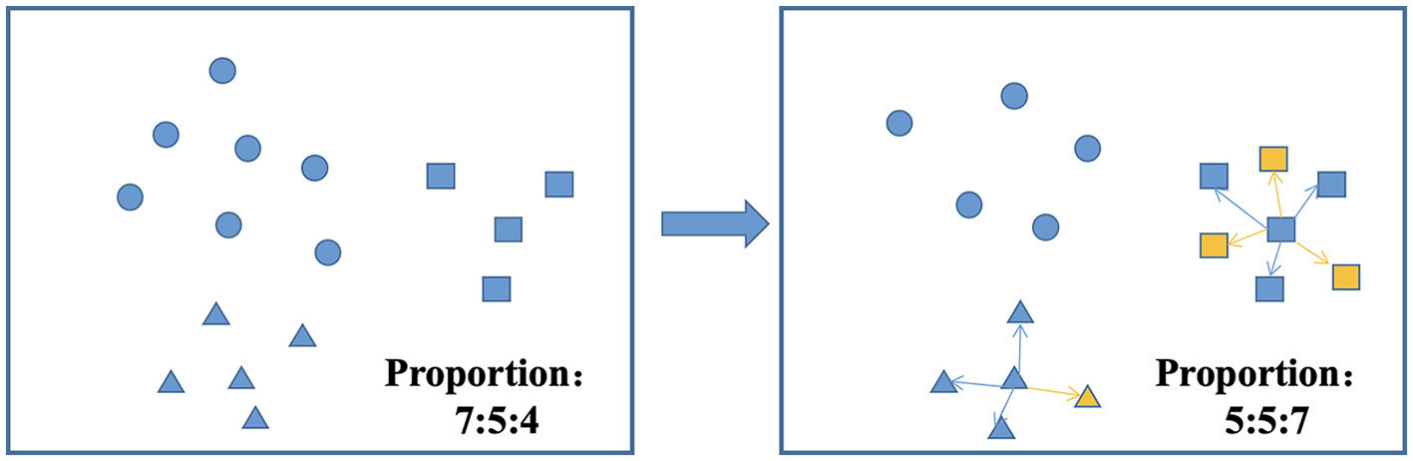
SMOTEENN balancing algorithm.

Fifth, there are three common approaches to dealing with class imbalance: undersampling, oversampling, and hybrid sampling techniques. Undersampling techniques include the random undersampling technique, and oversampling techniques include the random oversampling, SMOTE, adaptive synthetic (ADASYN), and borderline-SMOTE techniques (59). SMOTEENN is a method that combines oversampling and undersampling to handle both sample imbalance and noisy data. The SMOTE method increases the number of minority class samples by random oversampling, while the ENN method reduces the number of majority class samples by removing majority class samples. The combination of these two methods can better balance the class distribution in the dataset, thus improving the performance of the classifier (60, 61). The balancing algorithm can balance the number of samples for each classification, thus effectively improving the prediction performance of the model with unbalanced datasets (62, 63). Figure 4 shows the process of the SMOTEENN balancing algorithm, which not only synthesizes new samples for minority classes but also prunes duplicate samples to improve the difference between groups.

### Model construction and optimization

**Figure 5:**
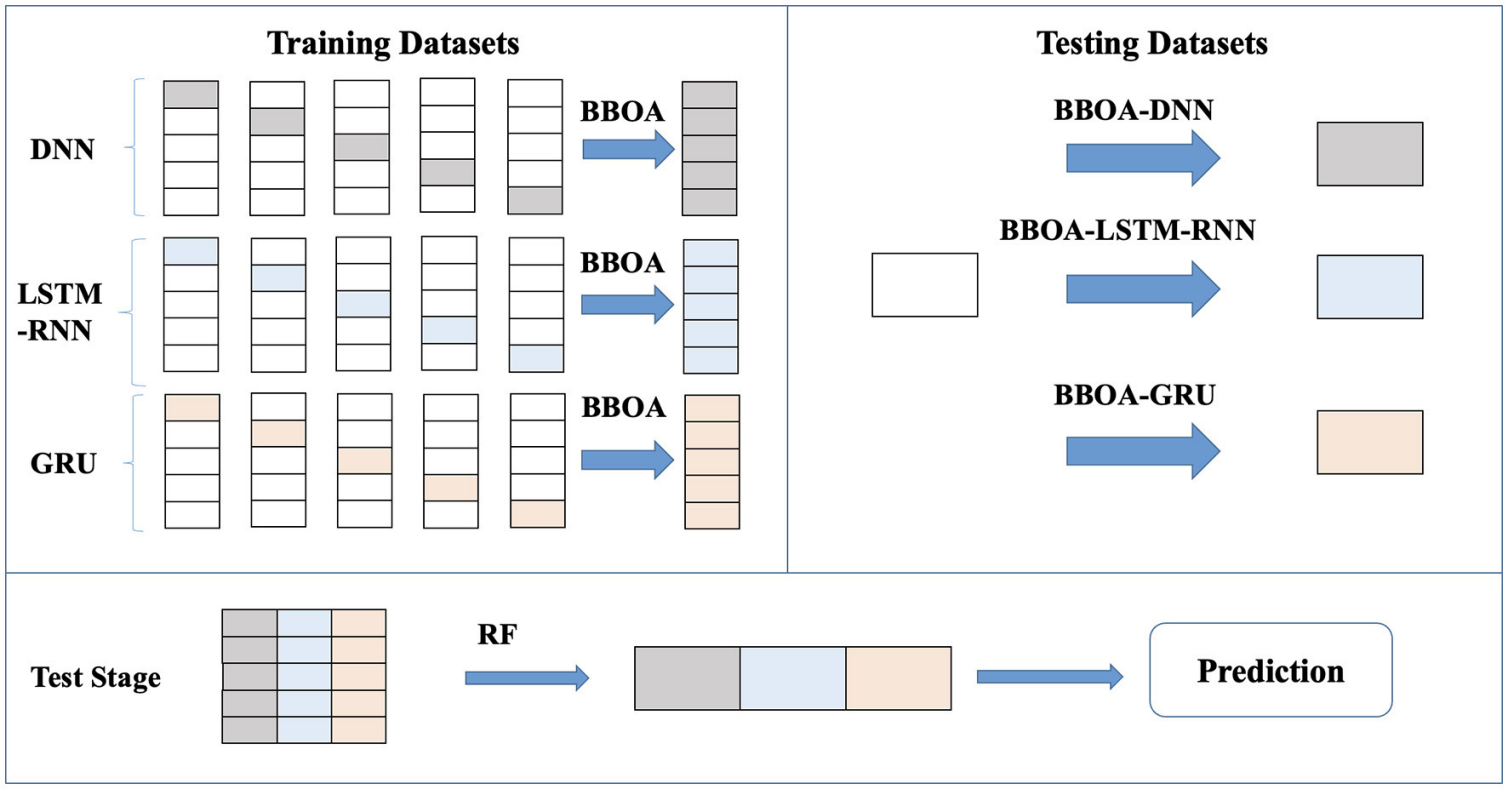
Construction process of the deep integration learning method.

The content in this section can be divided into the selection of the base learner, model construction, model optimization, and other steps. The construction process combines the ideas of ensemble learning and deep learning to construct an ensemble of deep learning (EDL) model with a multilayer cascade structure. The optimized ensemble of deep learning (OEDL) model is established by adding an optimization algorithm. The model is built as shown in Figure 5.

**Figure 6:**
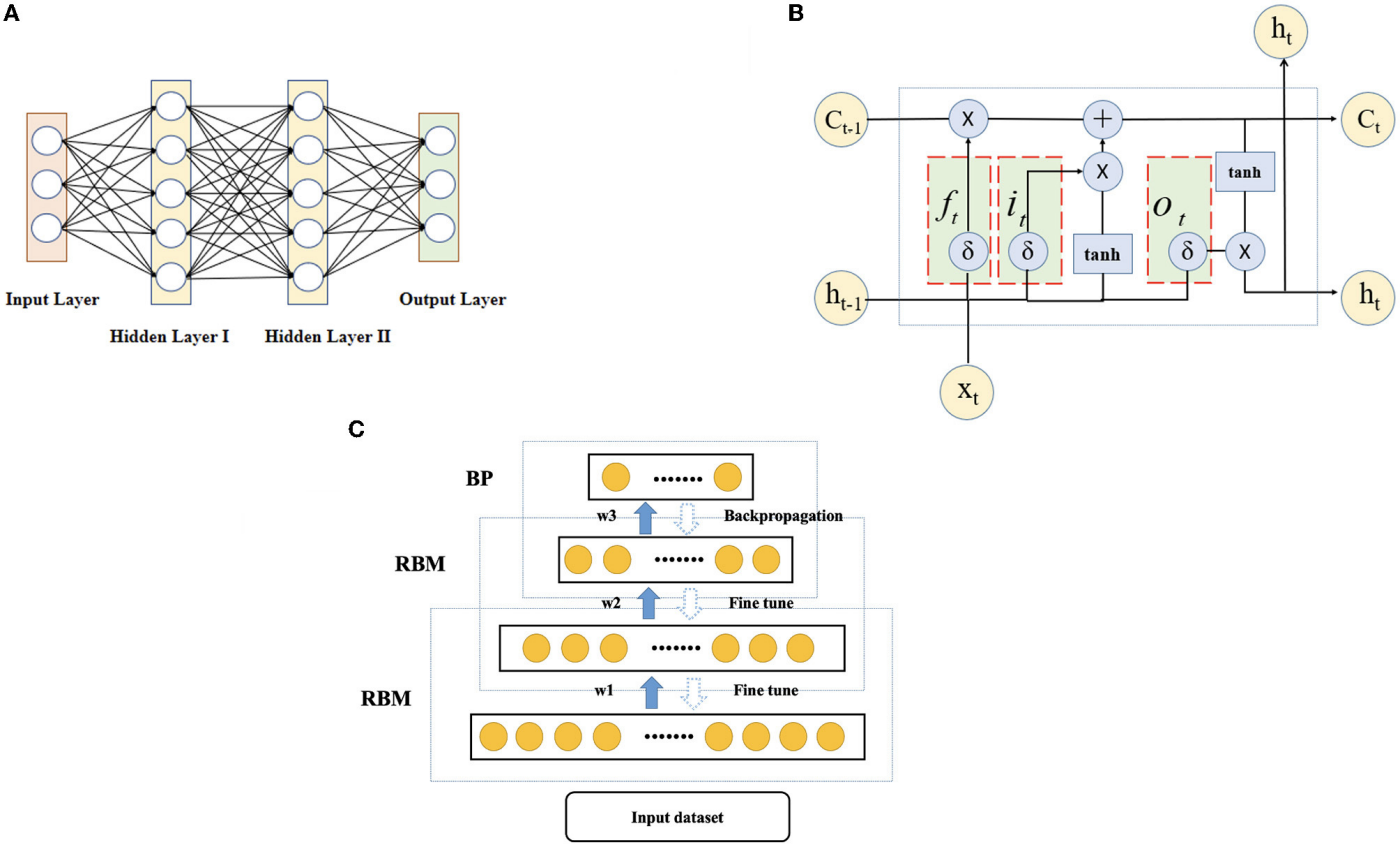
Schematic diagram of each base learner. **(A)** DNN, **(B)** LSTM-RNN, and **(C)** DBN.

First, the selection of base learners is needed. DNN, LSTM-RNN and DBN are used as base learners, and the schematic diagram of each base learner is shown in Figure 6. (A) A deep neural network (DNN) refers to a neural network with more than one hidden layer [64]. The input layer and hidden layer, hidden layer and hidden layer, and hidden layer and output layer all have linear relationships, which can be expressed as


(8) $$y_{i}=\sigma (\sum w_{i}^{n}x_{i}^{n}+b_{i})$$

where *y*_*i*_ is the next neuron, *x*_*i*_ is a feature or neuron connected to *y*_*i*_, σ is an activation function in a layer, *n* is the number of neurons or features connected to the neuron, *w*_*i*_ is a weight coefficient between a feature and a neuron or between neurons, and *b* is a constant. (B) Long short-term memory (LSTM) is proposed to solve the problem of vanishing or exploding gradients in recurrent neural networks (64, 65). The unit structure records the patient characteristic information of the current state by introducing a new internal state and carries out internal information transmission. First, an input gate *i*_*t*_, a forget gate *f*_*t*_, and an output gate *o*_*t*_ are calculated by using the patient characteristic information *x*_*t*_ of the current state and the hidden state *h*_*t*−1_ of the last time. Then, the input gate *i*_*t*_ and the forget gate *f*_*t*_ are used to control the retained historical characteristic information and the current state characteristic information of the patient, respectively, to obtain a new *C*_*t*_. Finally, the input gate *o*_*t*_ is used to transfer the patient characteristic information of the internal state to the hidden state *h*_*t*_. To achieve the classification effect, an RNN fully connected layer is added behind the LSTM unit to construct an LSTM-RNN to obtain a multi-classification result. (C) Deep belief networks (DBNs) are probabilistic generative models consisting of multiple layers of restricted Boltzmann machines. The main structure combines several layers of RBM and one layer of a BP network and outputs the results by the BP network. The specific steps are as follows. First, the features are trained in each layer of the RBM network separately in an unsupervised manner to ensure that the feature information is reused and retained. Then, the trained features enter the BP network to train the classifier through supervision. Finally, a backpropagation network fine-tunes the training error information direction of each RBM layer so that optimization can be achieved throughout the whole network. The parameters of the DBN are given by *w* (connection weights), *b* (visible unit bias), and *c* (hidden unit bias). The probability of input vector *v* and output vector *h* is given by


(9) $$\begin{array}{l} p\left(v,h\right)=\frac{e^{-E\left(v,h\right)}}{Z} \end{array}$$

where −*E*(*v, h*) is the energy function


(10) $$\begin{array}{l} E\left(v,h\right)=-b^{T}v-c^{T}h-h^{T}Wv \end{array}$$

*Z* is the normalizing factor obtained by summing the numerator over all possible statuses of *h* and *v*:


(11) $$\begin{array}{l} Z=\underset{v,h}{\sum }-E\left(v,h\right) \end{array}$$

Second, after considering bagging, boosting, stacking, and other methods (see the section Results), we chose the stacking algorithm as the ensemble method in this study, and the model constructed by it is named as the ensemble of deep learning (EDL). Stacking is a method that combines the outputs of multiple base learners according to a certain combination strategy (66, 67). We chose classical and representative deep learning models such as DNN, LSTM-RNN, and DBN as the base learner (68–70) and random forest (RF) as the meta-learner. After the training of each base learner was completed, we used the stacking algorithm for analysis; that is, the outputs of multiple base learners were taken as a new dataset that was incorporated into the meta-learner (random forest was selected in this study) for learning and prediction. We integrated the results of the three neural networks and formed probabilities for the three classifications to obtain the final prediction for each sample. The deep learning system was iterated 100 times, and finally, the optimal model was selected by using a greedy strategy. The pseudocode for the stacking algorithm is shown in Algorithm 1.

**Algorithm 1:** Stacking pseudocode.


1: **————————Step 1: Input**
Dataset D = {(x_1(1)_, x_2(1)_, ..., y_(1)_), (x_1(2)_, x_2(2)_, ..., y_(2)_), (x_1(n)_, x_2(n)_, ..., y_(n)_)}; Primary learning algorithm : PLA = {DNN, LSTM-RNN, DBN}; Secondary learning algorithm:Random Forest(RF) 3: **———————-Step 2:** **Process** 1: **split** D:Train_data, Testing_data 2: **for** t = 1, 2, ..., T **do** 3: h_(t)_ = *Stratified Fold* (Train_data); 4: **end for** 5:New_Train_data =Ø; 6: **for** *i* in PLA **do** 7: **for** t = 1, 2, ..., T **do** 8: *Z*_*it*_ =h_(t)_(*PLA*__(*i*)__); 9: **end for** 10: New_Train_data = ∪((*Z*_*i*1_, *Z*_*i*1_, ..., *Z*_*i*1_), *y*_*i*_); 11: **end for** 12: New_Test_data = Ø; 13: **for** *i* in PLA **do** 14: **for** t = 1, 2, ..., T **do** 15: *Z*_*it*_= h_(t)_(*PLA*__(*i*)__); 16: **end for** 17: New_Test_data = ∪ ((*Z*_*i*1_, *Z*_*i*1_, ..., *Z*_*i*1_), *y*_*i*_); 18: **end for** 19: Training_RF = RF(New_Train_data) 3: **———————Step 3: Output** Testing_RF = Training_RF (h_(1)_(New_Test_data_(1)_), h_(2)_(New_Test_data_(1)_), ..., h_(T)_(New_Test_data_(1)_))

The innovations of the OEDL method proposed in this study can be reflected in the following aspects. (A) When splitting the training set and the test set, the random stratification method is improved to the label percentage stratification approach to achieve the effect of label balancing. (B) During data selection, we selected clinical features and radiomics features in turn, analyzed clinical features and high-dimensional, abstract radiomics information as combined features, and finally built a combined feature model. (C) We chose the method of deep integration and comprehensively utilized the advantages of each deep learning model to improve its effectiveness and generalization. (D) We innovatively used an improved metaheuristic algorithm (see Section Model training for details) for optimization purposes to ensure the excellence of the classification results.

**Figure 7:**
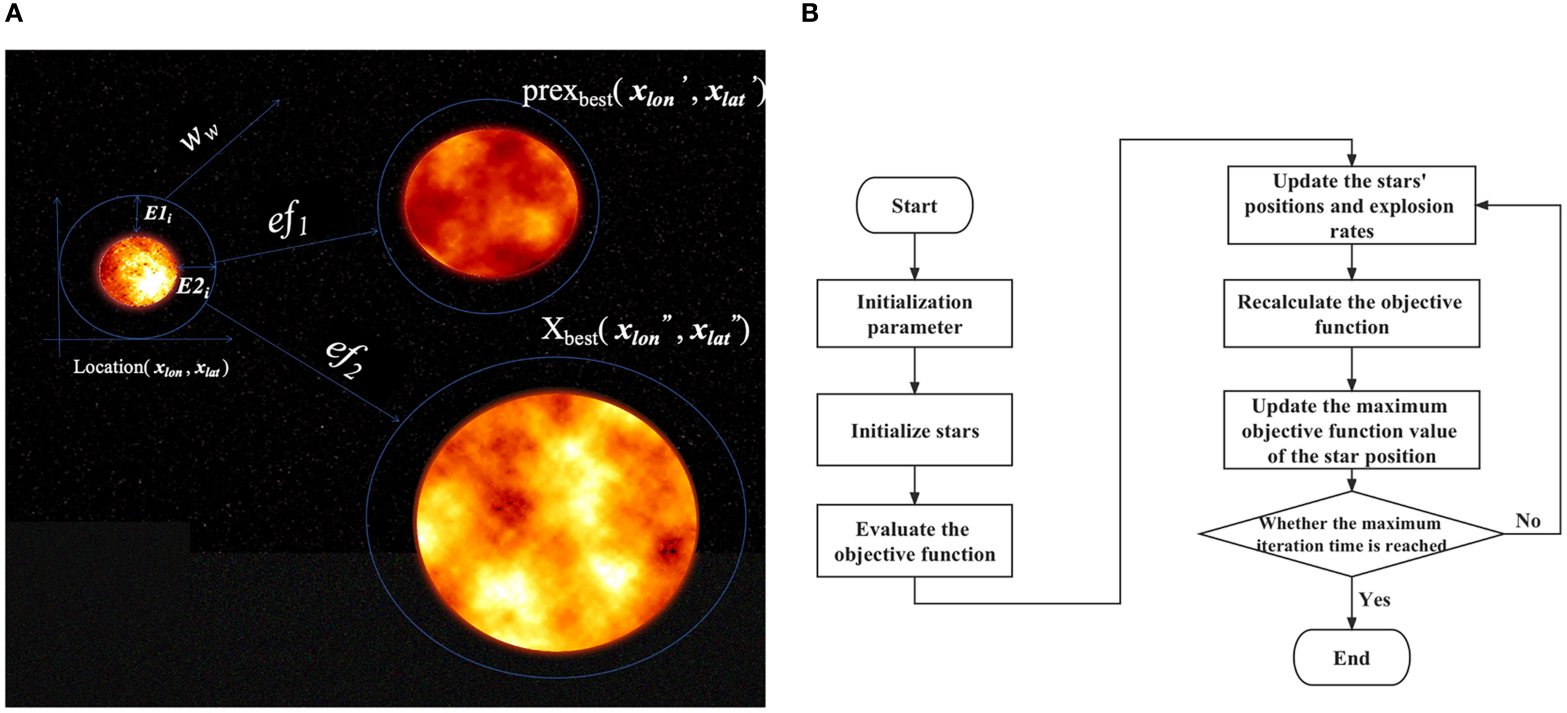
BBOA schematic diagram. To solve the parameter optimization problem faced by deep networks, we used the Big Bang optimization algorithm (BBOA). **(A)** The concept of a cosmic explosion; **(B)** the BBOA pipeline (the PUOA algorithm procedure).

Third, we performed model optimization based on a newly proposed optimization algorithm. The above EDL proposal combines deep learning and ensemble learning ideas, but there is still the problem of the slow hyperparameter search. To solve this problem, we considered introducing a metaheuristic algorithm. Based on the stacking idea and the framework of particle swarm optimization, we proposed the big bang optimization algorithm (BBOA), which aims to solve the parameter optimization problem in deep networks and applied it to the OEDL method. During the analysis, the algorithm draws on the particle swarm optimization algorithm and the black hole theory of the Big Bang (71), as shown in Figure 7. In the process of constructing the algorithm, we used a sinusoidal chaotic map, an adaptive inertia weight, a greedy strategy, and other optimization methods. The symbol descriptions of the algorithm are shown in Table 1.

**Table 1:** Algorithm process symbol description.

**Notation**	**Meaning**
${x_{j}}^{H}$	Initial position of the *j*-th galaxy (horizontal axis)
${x_{j}}^{L}$	Initial position of the *j*-th galaxy (vertical axis)
$m_{ij}{\;}^{H}$	Initial position of the *i*-th star in the *j*-th galaxy (horizontal axis)
$m_{ij}{\;}^{L}$	Initial position (vertical axis) of the *i*-th star in the *j*-th galaxy
*U*_*x*_	Upper boundary of the galaxy (horizontal axis)
D_x_	Lower boundary of the galaxy (vertical axis)
ef_1_	The speed of the fastest star in the galaxy
ef_2_	The speed of the fastest star in the universe
*r*	Random number between 0 and 1
${E_{j}}^{H}$	Galactic expansion speed (horizontal axis)
${E_{j}}^{L}$	Expansion speed of the galaxies (vertical axis)
${{\text{H}}_{\text{i}}}^{\text{t}}$	Time position of the largest star t in the galaxy (horizontal axis)
${{\text{L}}_{\text{i}}}^{\text{t}}$	Time position of the largest star t in the galaxy (vertical axis)
${\text{ef}}_{\text{1}}$	The speed of the fastest star in the galaxy
${\text{ef}}_{\text{2}}$	The speed of the fastest star in the universe
*w*_*s*_	The initial inertia of stars in the galaxies
*w*_*e*_	The final inertia of stars in the galaxies
*t*	Evolution time (number of iterations)
*T*	Total evolution time (iterations)
*w*_*w*_	Adaptive inertia weight
Y_o_	Velocity of the largest star in the universe (optimal solution)
*y*_*o*_	The velocity of the largest star in the galaxy

(A) The galaxy's initial position can be expressed as


(12) $$x_{j}^{\;\;H}=U_{x}+m_{ij}^{\;\;H}*(D_{x}-U_{x})$$

(13) $$x_{j}^{\;\;L}=U_{x}+m_{ij}^{\;\;L}*(D_{x}-U_{x})$$

(14) $$m_{ij}^{\;\;H}=a{\left(m_{i-1j}^{\;\;\;\;\;\;H}\right)}^{2}\sin(\pi m_{i-1j}^{\;\;\;\;\;\;H})$$

(15) $$m_{ij}^{\;\;L}=a{\left(m_{i-1j}^{\;\;\;\;\;\;L}\right)}^{2}\sin(\pi m_{i-1j}^{\;\;\;\;\;\;L})$$

In the study, Formula (14) and Formula (15) add the sinusoidal chaotic map, which acts as an initial randomization to make the distribution range of the star group more dispersed (72). A sinusoidal chaotic map is a non-linear map that can produce chaotic phenomena (73), where *a* is any constant, and the initial values $m_{ij}{\;}^{H}$ and $m_{ij}{\;}^{L}$ can be any number, but for the depth rule suitable for the deep learning neural network, the initial value is a random integer between 0 and 100, and the chaotic map is rounded to indicate the number of neurons in the deep learning.

(B) The rates of expansion of the galaxies can be expressed as


(16) $$E_{i}^{\;H}=w_{w}E_{1i}^{\;t-1}+ef_{1}\cdot r(y_{oi}^{\;t}-H_{i}^{\;t})+ef_{2}\cdot r(y_{oi}^{\;t}-H_{i}^{\;t})$$

(17) $$E_{i}^{\;L}=w_{w}E_{2i}^{\;t-1}+ef_{1}\cdot r(y_{oi}^{\;t}-L_{i}^{\;t})+ef_{2}\cdot r(y_{oi}^{\;t}-L_{i}{\;}^{t})$$

(18) $$w_{w}=w_{s}\text{ - }(w_{s}\text{ - }w_{e})\frac{t}{T}$$

In the study, Formula (18) is the added adaptive inertia weight, and its function is to regulate the initial expansion speed. Adaptive inertia weights are a variant of inertia weights. Each galaxy should constantly consider its historical and global best position when updating its expansion speed. The adaptive inertia weight can dynamically adjust the value of the inertia weight according to the historical state of the galaxy so that the algorithm converges to the optimal solution faster. The function of the adaptive inertia weight is to regulate the initial expansion speed.

(C) The transformation of the production expansion center of galaxies affected by higher expansion velocities is expressed as


(19) $$\begin{array}{l} x{\left(\text{H}\right)}_{i}=\text{x}{\left(\text{H}\right)}_{i}+{{\text{E}}_{\text{i}}}^{H} \end{array}$$

(20) $$\begin{array}{l} x{\left(\text{L}\right)}_{\text{i}}=\text{x}{\left(\text{L}\right)}_{\text{i}}+{{\text{E}}_{\text{i}}}^{L} \end{array}$$

(D) The optimal solution is the velocity of the largest star in the universe, which can be expressed as


(21) $$Y_{o}=\{\begin{array}{cc} y_{o}, & ify_{o}>Y_{o};\\ Y_{o}, & else. \end{array}$$

In this study, to update the optimal solution, the greedy strategy is used, which can be expressed as


(22) $$\begin{array}{l} y_{o}=\text{max}\left({y_{o}}^{t}\right) \end{array}$$

BBOA was used in OEDL to optimize the number of hidden layers and the number of neurons in each layer of the DNN, LSTM-RNN, and DBN. The stacking integration algorithm was used to integrate the three models after each model was optimized.

### Model training

This research was carried out on a Linux workstation equipped with a GPU. The software platform was based on Python 3.7. The proposed algorithms were implemented based on the TensorFlow 2.8 framework. The GPU was used to accelerate the training process. Among the study population, 70% of the data were randomly selected for the training set, and the remaining 30% were used as the test set. Statistical analysis was performed using Python 3.7.0, SPSS 26.0 (SPSS Inc., Chicago, IL, USA).

For various algorithm models, the algorithm was implemented using data split based on a ratio of 7:3 for training and testing. The model was fitted with the training set, the hyperparameters except for the number of hidden layers and the number of neurons were determined by the grid search method, and the best parameter model was selected after 50% cross-validation. Finally, the model was tested with the test set to evaluate the generalizability of each model. The hyperparameters determined by the grid search method in this study were based on a learning rate of 10-4, a batch size of 20, a momentum term of 0.9, and 1,000 epochs. In addition, the imbalanced distribution of the sample size in each category will lead to the prediction bias of the model. To eliminate this effect, we used the SMOTEENN algorithm to enhance the fused features.

In this study, the implementation of the deep learning network was based on the Keras package in TensorFlow 2.8. The Adam optimizer was used to optimize the gradient of the deep learning model, and the cross-entropy loss function was combined with the softmax activation function to obtain better classification results. The neural network of the three base learners was initially set as a double layer, and the number of neurons in each layer was 10. The BBOA optimizes the numbers of layers and neurons of each base learner to fix the model.

The evaluation indicators in the classification model were obtained based on a confusion matrix, and they include the following four basic indicators: “true positive” (TP) means that the prediction is true and the actual value is also true; “true negative” (TN) means that the prediction is false and the actual value is also false; “false positive” (FP) means that the prediction is true but the actual value is false; “false negative” (FN) means that the prediction is false but the actual value is true. Among multiple classes, each class *i* has values TP_i_, TN_i_, FP_i_, and FN_i_. T_i_P_i_ represents that the true class *i* is correctly predicted as class *i*, and *F*_j_P_i_ represents that the true class *j* is incorrectly predicted to be class *i*.

In this study, the evaluation indices include Macro-AUC, accuracy (ACC), macrosensitivity (Macro-R), macrospecificity (Macro-P), and Macro-F1 score (Macro-F1) (74). The ROC curve for each classification was plotted with the true positive rate of each classification as the vertical axis and the false positive rate of each classification as the horizontal axis. The area under the ROC curve of each category is the AUC value of each category, and Macro-AUC is the sum of all types of areas and the average. The value range is [0 ~ 1]. The greater the value is, the more accurate the classification. The indicators can be expressed as follows:


(23) $$\begin{array}{l} \text{ACC}=\frac{\overset{\text{n}=3}{\underset{\text{i}=1}{\sum }}\text{T}{\text{P}}_{\text{i}}}{\overset{\text{n}=3}{\underset{\text{i}=1}{\sum }}\left(\text{T}{\text{P}}_{\text{i}}+\text{F}{\text{P}}_{\text{i}}\right)} \end{array}$$

(24) $$\begin{array}{l} \text{macro\_}\text{PRE}=\frac{1}{\text{n}}\overset{\text{n}=3}{\underset{\text{i}=1}{\sum }}\left(\frac{\text{T}{\text{P}}_{\text{i}}}{\text{T}{\text{P}}_{\text{i}}+\text{F}{\text{P}}_{\text{i}}}\right) \end{array}$$

(25) $$\begin{array}{l} \text{macro\_}\text{SEN}=\frac{1}{\text{n}}\overset{\text{n}=3}{\underset{\text{i}=1}{\sum }}\left(\frac{\text{T}{\text{P}}_{\text{i}}}{\text{T}{\text{P}}_{\text{i}}+\text{F}{\text{N}}_{\text{i}}}\right) \end{array}$$

(26) $$\begin{array}{l} macro\text{\_}F1\text{score}=\frac{2\cdot macro\text{\_}PRE\cdot macro\text{\_}SEN}{macro\text{\_}PRE\;+\;macro\text{\_}SEN} \end{array}$$

## Results

The results include the results of clinical feature selection, imaging feature selection, comparison of the prediction performance of each method, comparison of the prediction performance of each feature, comparison of the prediction performance of each balanced method, and comparison with previous studies.

### Results of clinical feature selection

**Figure 8:**
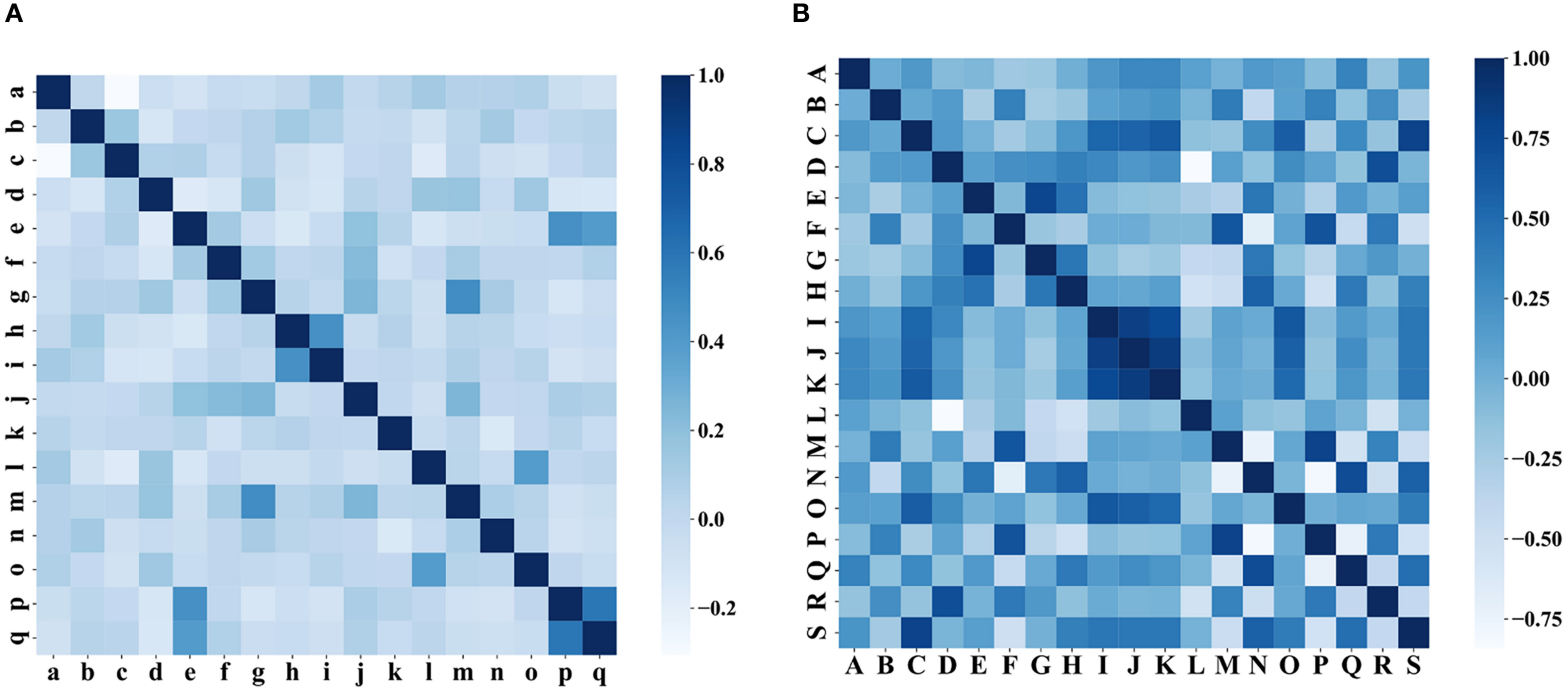
Correlation test of characteristics. **(A)** Correlation test of clinical characteristics and **(B)** correlation test of the iconographic features.

In the clinical feature selection stage, 17 features were included in the model, and all of these features passed the correlation test (as shown in Figure 8). The clinical characteristics according to the discharge NIHSS classification are presented in Table 2.

**Table 2:** Clinical characteristics according to the discharge NIHSS category.

**Number**	**Clinical factors**		**Group A (*n* = 106)**	**Group B (*n* = 289)**	**Group C (*n* = 46)**
a	NIHSS on admission		0 (0, 1)	2 (1, 3)	7 (5, 9)
b	Position	Telencephalon	53 (50.00%)	122 (42.21%)	22 (47.83%)
		Diencephalon	15 (14.15%)	91 (31.49%)	15 (32.61%)
		Cerebellum	23 (21.70%)	5 (1.73%)	0 (0.00%)
		Brain stem	15 (14.15%)	71 (24.57%)	9 (19.57%)
c	OCSP typing	TACI	0 (0.00%)	2 (0.69%)	9 (19.57%)
		PACI	22 (20.75%)	116 (40.14%)	26 (56.52%)
		LACI	44 (41.51%)	79 (27.34%)	10 (21.74%)
		POCI	40 (37.74%)	92 (31.83%)	1 (2.17%)
d	Age		67.17 ± 11.63	66.92 ± 12.09	65.63 ± 14.82
e	Sex	Male	66 (62.26%)	167 (57.79%)	24 (52.17%)
		Female	40 (37.74%)	122 (42.21%)	22 (47.83%)
f	BMI		24.29 ± 3.10	24.13 ± 3.94	25.29 ± 4.15
g	SBP		160.79 ± 14.96	162.90 ± 13.97	161.85 ± 18.70
h	TC		4.48 ± 1.05	4.60 ± 1.11	4.38 ± 1.14
i	LDL		2.61 ± 0.92	2.70 ± 1.01	2.64 ± 1.14
j	LVH	Yes	61 (57.55%)	161 (55.71%)	31 (67.39%)
		No	45 (42.45%)	128 (44.29%)	15 (32.61%)
k	HCY	Yes	10 (9.43%)	30 (10.38%)	6 (13.04%)
		No	96 (90.57%)	259 (89.62%)	40 (86.96%)
l	AF	Yes	9 (8.49%)	21 (7.27%)	10 (21.74%)
		No	97 (91.51%)	268 (92.73%)	36 (78.26%)
m	Hypertension	Yes	93 (87.74%)	265 (91.70%)	43 (93.48%)
		No	13 (12.26%)	24 (8.30%)	3 (6.52%)
n	Diabetes	Yes	35 (33.02%)	124 (42.91%)	23 (50.00%)
		No	71 (66.98%)	165 (57.09%)	23 (50.00%)
o	CHD	Yes	6 (5.66%)	17 (5.88%)	6 (13.04%)
		No	100 (94.34%)	272 (94.12%)	40 (86.96%)
p	Smoking	Yes	32 (30.19%)	62 (21.45%)	13 (28.26%)
		No	74 (69.81%)	227 (78.55%)	33 (71.74%)
q	Drinking	Yes	25 (23.58%)	52 (17.99%)	8 (17.39%)
		No	81 (76.42%)	237 (82.01%)	38 (82.61%)

Groups A, B, and C refer to the normal group, mild group, and moderate-severe group, respectively. SBP, systolic blood pressure; TC, total cholesterol; LDL, low-density lipoprotein; LVH, left ventricular hypertrophy; HCY, homocysteinemia; AF, atrial fibrillation; CHD, coronary heart disease; TACI, OCSP classification, total anterior circulation infarcts; PACI, partial anterior circulation infarcts; LACI, lacunar circulation infarcts; and POCI, posterior circulation infarcts.

### Results of radiomics feature selection

**Figure 9:**
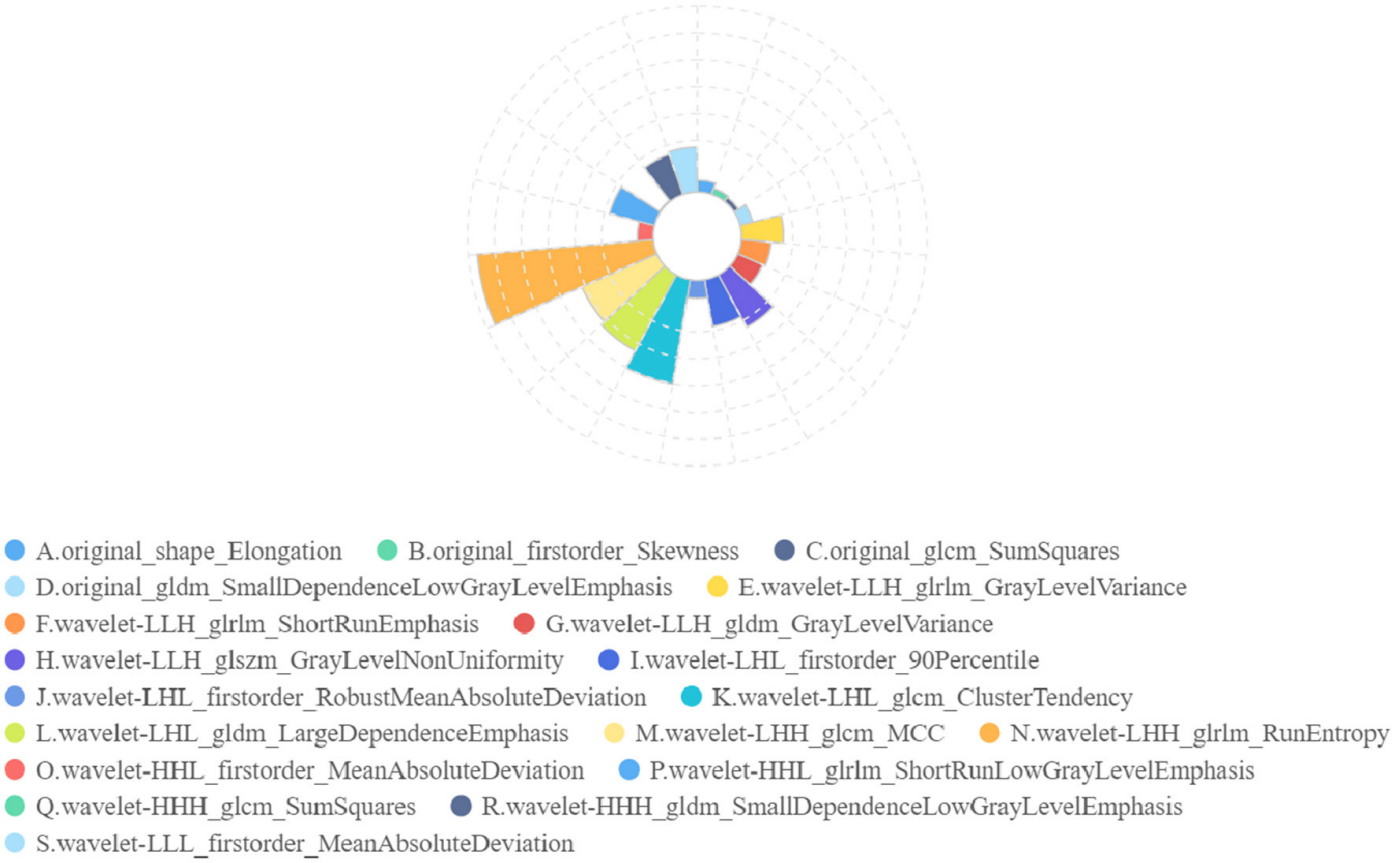
Rose plot of feature weights. The 19 extracted features are represented by A to P, and the feature weights are shown.

In radiomics feature selection, we first selected 328 features from 851 features using variance selection (threshold 0.3), used the LightGBM and XGBoost algorithms to screen out 81 more important features with the top 50 weights, and sorted out 19 features that appeared in both methods and the top 10 features in their respective methods. After the correlation test, 19 image features were selected. A rose plot was drawn based on the 19 features and their importance weights to the model, as shown in Figure 9.

**Figure 10:**
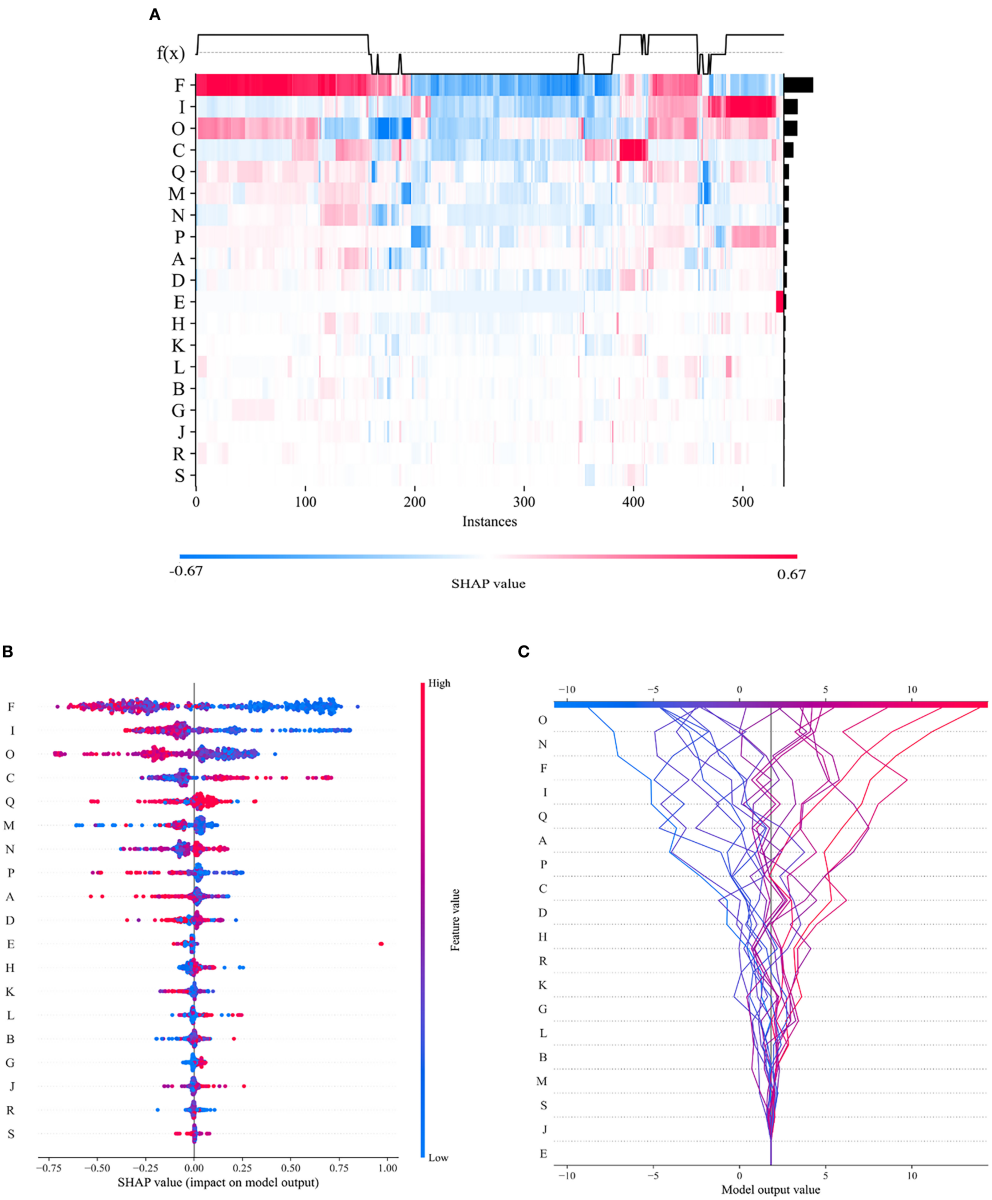
Visual interpretation of the importance of selected features. **(A)** Feature density scatterplot: each column represents a sample, and each row represents a feature; the features are sorted by their average absolute SHAP values; red represents the positive direction, and blue represents the negative direction. **(B)** Feature distribution heatmap: each point represents a sample, the samples are sorted by their SHAP values, and the absolute SHAP value of a feature represents its contribution to the model. **(C)** Feature decision diagram: this figure represents the accumulation of all samples and features as well as model's decision-making process.

To visualize the importance of the selected features, we used SHAP to illustrate the degrees to which these features influenced the prediction results, as shown in Figure 10. The SHAP value represents the contribution of each feature to the final prediction and can effectively explain the model prediction for each sample. The feature ranking (y-axis) represents the importance of the prediction model, and the corresponding SHAP value (x-axis) represents the degree of feature influence.

### Comparison of the prediction performance of each method

Using the joint dataset as an example, the classification results of these methods are compared. The results show that the OEDL constructed based on the concept of ensemble optimization obtained the best classification performance, and its Macro-AUC, ACC, Macro-R, Macro-P, and Macro-F1 reached 97.89, 95.74, 94.75, 94.03, and 94.35%, respectively, as shown in Table 3.

**Table 3:** Comparison of the classification effects of different methods (%).

**Type**	**Name**	**Macro-AUC**	**ACC**	**Macro-R**	**Macro-P**	**Macro-F1**
Machine learning	DT	90.53	75.53	59.62	50.37	54.41
	SVM	97.69	82.45	72.56	82.71	74.11
	RF	95.90	87.77	79.67	85.28	81.14
Deep learning	DNN	93.13	82.96	83.26	83.02	83.12
	LSTM-RNN	94.56	84.81	84.81	84.66	84.63
	DBN	94.39	83.70	83.70	83.61	83.61
Deep learning + Ensemble learning	Hard-voting	95.36	87.23	86.86	87.01	86.90
	Soft-voting	93.45	87.23	86.86	87.10	86.92
	EDL	96.68	92.55	92.10	91.42	91.72
OEDL	OEDL	**97.89**	**95.74**	**94.75**	**94.03**	**94.35**

EDL represents a deep ensemble learning model based on DNN, LSTM-RNN, DBN, and stacking ensemble; OEDL is an optimization algorithm based on EDL and BBOA.

These bold characters represent the predictive performance of the optimal method.

### Comparison of the prediction performance of each feature

**Figure 11:**
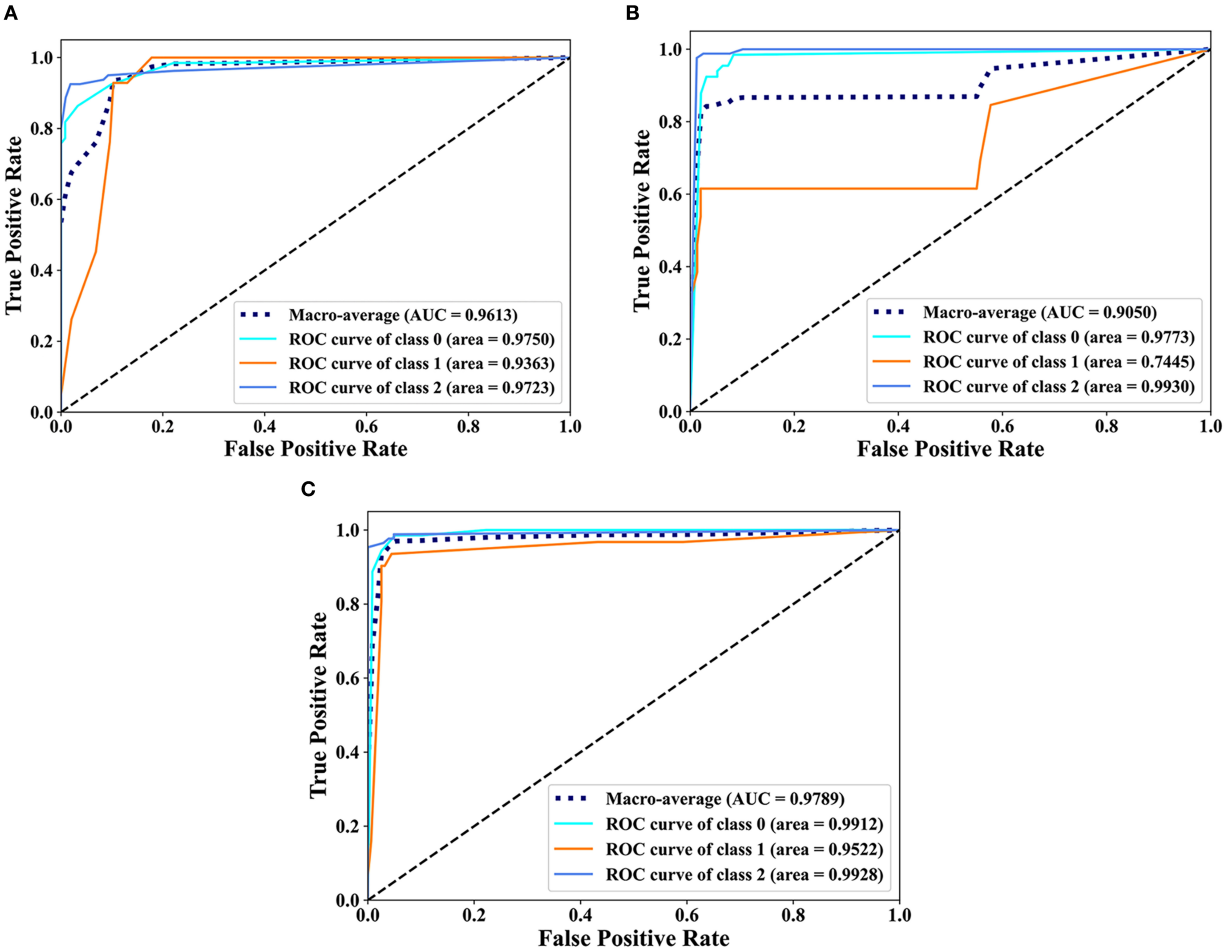
Scatter plot display of the classification results of OEDL. **(A)** Clinical, **(B)** Radiomics, and **(C)** Joint.

The classification results of EDL and OEDL were compared. The results show that compared with that using the clinical and radiomics features, the method using the combined data had better classification performance, and the EDL method achieved a Macro-AUC of 96.68% and an ACC of 92.55%. The OEDL method achieved a Macro-AUC of 97.89% and an ACC of 95.74%, as shown in Table 4. We also visualized the classification results of the OEDL method with the three features in the form of ROC curves, as shown in Figure 11.

**Table 4:** Comparison of classification performance of various feature combinations (%).

**Feature**	**Model**	**Macro-AUC**	**ACC**	**Macro-R**	**Macro-P**	**Macro-F1**
Clinical	EDL	97.15	88.30	87.60	86.78	86.82
Radiomics	EDL	90.79	90.74	74.10	80.28	75.82
Joint	EDL	96.68	92.55	92.10	91.42	91.72
Clinical	OEDL	96.13	90.43	90.57	89.29	89.35
Radiomics	OEDL	90.50	93.21	82.19	86.27	83.87
Joint	OEDL	**97.89**	**95.74**	**94.75**	**94.03**	**94.35**

EDL represents a deep ensemble learning model based on DNN, LSTM-RNN, DBN, and stacking ensemble; OEDL is an optimization algorithm based on EDL and BBOA.

These bold characters represent the predictive performance of the optimal method.

### Comparison of the prediction performance of each balanced method

The classification results of the combined features and the OEDL method were compared. Compared with the unbalanced, oversampled, and undersampled techniques, SMOTEENN based on a mixed sampling method achieved the best classification performance, and its Macro-AUC, ACC, Macro-R, Macro-P, and Macro-F1 reached 97.89, 95.74, 94.75, 94.03, and 94.35%, respectively, as shown in Table 5.

**Table 5:** Comparison of the classification performance of various balancing methods (%).

**Method**	**Type**	**Macro-AUC**	**ACC**	**Macro-R**	**Macro-P**	**Macro-F1**
Original	None	92.60	81.16	85.01	74.62	78.75
Random Oversample	Oversampling	91.84	80.37	80.37	81.14	80.49
Random Undersample	Oversampling	82.78	73.81	71.82	74.52	72.47
SMOTE	Undersampling	90.98	85.93	86.10	85.85	85.85
ADASYN	Undersampling	95.74	84.84	85.05	85.35	84.28
Borderline-SMOTE	Undersampling	96.10	87.04	87.12	87.68	86.75
SMOTEENN	Mixed-sampling	97.89	95.74	94.75	94.03	94.35

### Comparison with previous studies

To demonstrate the advanced performance of the method proposed in this study, we reviewed relevant research in the field of AIS classification and prediction and compared the AUC and ACC of each study. Although the datasets, classification numbers, and other aspects of these studies differed, the differences in the results have some implications for the excellence of the methods. The comparison results show that the proposed method has significantly better classification performance in terms of AUC, ACC, and other aspects than that of previous studies, and it has better classification advantages. For more information, see Table 6.

**Table 6:** Comparison of classification performance with previous studies.

**Authors**	**Data**	**Methods**	**Number of categories**	**AUC (%)**	**ACC (%)**
Qiu (16)	Radiomics	SVM	2	85	-
Alaka (17)	Clinical	LR + Machine Learning	2	66–71	-
Hofmeister (26)	Clinical + Radiomics	SVM	2	88.1	-
Wang (24)	Clinical + Radiomics	LR	2	73	-
Ours	Clinical + Radiomics	OEDL	3	**97.89**	**95.74**

These bold characters represent the predictive performance of the optimal method.

## Discussion

AIS is one of the many diseases that endangers the health of Chinese residents. It is difficult and expensive to check, and it is difficult to evaluate the early prognosis (75). We used joint features to train the OEDL model to predict the prognosis of AIS, which is of great significance to improve the diagnosis and prevention system of AIS and promote the optimal allocation of medical resources.

In terms of data collection and processing, we used clinical features and radiomics features creatively to build joint features, and we built a complete and feasible data processing operation process. Compared with the clinical and imaging feature models, the method using the combined data had better classification performance, and the EDL method achieved a Macro-AUC of 96.68% and an ACC of 92.55%. The OEDL method obtained a Macro-AUC of 97.89% and an ACC of 95.74%. Joint-feature modeling produces better results than single-feature modeling. The reason for the analysis is that simple clinical feature model information is easy to collect, but prediction efficiency is limited due to clinical feature instability; radiomics features can be used to achieve high prediction efficiency, but the inclusion of purely influencing omics features is limited; joint feature modeling can incorporate more comprehensive and objective information. Feature selection is the process of finding the feature subset that yields the best model performance, which is conducive to removing redundant features and avoiding the risk of overfitting. Our data collection and processing techniques can be actively promoted in future radiomics research.

Traditional methods for predicting AIS prognosis are shallow and deep machine learning methods. Their ability to represent complex problems is limited, as is their learning ability. To design a new OEDL and apply it to the prediction of AIS prognosis, we creatively combined the ideas of deep learning, integrated learning, and metaheuristic optimization. A comparison of the prediction performance of the various methods shows that the best classification performance was obtained by OEDL based on ensemble optimization, with Macro-AUC, ACC, Macro-R, Macro-P, and Macro-F1 reaching 97.89, 95.74, 94.75, 94.03, and 94.35%, respectively. The main reasons can be analyzed as follows. (1) In complex problems, the deep learning model can outperform the traditional shallow learning model in terms of feature learning ability. Deep learning multilayer networks can effectively represent the complexity of prediction results and are adept at discovering complex relationships between a large number of input features, resulting in high prediction performance (76). (2) When compared to a single learner, the advantage of integrated learning is that it ensures classifier diversity and richness, as well as better prediction effect and stability through stacking combination (77). (3) We developed a new parameter optimization strategy based on the traditional metaheuristic algorithm to address the problem of superparameter optimization in machine learning algorithms. Our optimization algorithm can effectively avoid the problem that traditional optimization methods have of falling into a local optimal solution, and it can also effectively improve the model efficiency (78).

In deep learning, the quality and quantity of data have a crucial impact on the training effect of the model. If the training data are imbalanced, i.e., the number of samples in some classes is too small, then the model will be biased toward those classes with a high proportion during the training process and will perform poorly for those classes with a low proportion. This results in poor model performance with test data may lead to overfitting. Therefore, we introduced a data balancing method to ensure the balance of the training data. The classification results of various balance methods were compared. Compared with the unbalanced, oversampled, and undersampled techniques, SMOTEENN based on mixed sampling can achieve the best classification performance. The results suggest that SMOTEENN, which combines undersampling and oversampling, is the most suitable balancing technique for this study.

A comparison with actual scenarios can be described as follows. Qiu (16) used a linear SVM method to analyze the optimal imaging group thrombus characteristics of IV protease recanalization with AIS patients on noncontrast CT (NCCT) and CT angiography and obtained (0.85 ± 0.03) ACC in the comparison of actual data. Multiple regression and machine learning models were used by Alaka (17) to predict the related dysfunction of AIS patients after intravascular therapy. Using an internal dataset, the model had an AUC of 0.65–0.72, and using an external dataset, the model had an AUC of 0.66–0.71. Hofmeister (26) investigated the predictive value of radiomics features extracted from clots on the first thrombosis recanalization using SVM, with an ACC of 0.88. Wang (24) obtained an ACC of 0.73 by using the modified Rankin scale (mRS) to predict the prognosis of AIS. Traditional methods for complex problems have limited expression and learning ability, so it is necessary to design a deep integration model with a multilevel cascade structure to improve the model's learning ability in complex problems (35). When compared to that of other single and integrated methods, OEDL can achieve the best classification performance when compared to the control method. The proposed method outperformed previous methods in terms of classification performance (AUC, ACC, etc.) and classification superiority. Furthermore, to address the problem of poor interpretability that frequently exists in deep learning (79), we used interpretable machine learning technology to understand the model's applicability to clinical prediction, with the goal of revealing the reasons behind the prediction results.

In a clinical sense, the combined feature model we developed can serve as a reliable clinical diagnostic tool for predicting stroke prognoses. Our modeling method is more suitable for the clinical model application scenario; it is convenient for radiologists to understand the differences between clinical features, morphological features, and high-dimensional omics features, as well as diagnostic performance differences. In addition, when building the training set, we also built a data validation set and performed in-model validation at a single center. This study confirmed the validity and scientific nature of the combined data, provided an important reference for similar subsequent studies, and facilitated further verification through the use of more external multi-center data. Compared with traditional radiomics analysis, our combined feature model could extract more statistical features, thereby providing a comprehensive stroke description. In addition, computerized tools overcome the instability of human empirical judgments, allowing clinicians to quickly and accurately predict long-term outcomes.

This study still has room for improvement. First, this study is a single-center, retrospective study with a limited sample size, and it is expected that a multi-center study with larger samples will be implemented in future to further verify the generalizability of the model. Second, lesion labeling comes from manual delineation and may be affected by the subjective judgment of investigators. Subsequent semiautomatic or fully automatic labeling algorithms need to be further explored to improve the stability and consistency of feature extraction. Third, the radiomics features constructed in this study are based on noncontrast-enhanced MR only, requiring further advanced MR acquisitions such as contrast-enhanced DWI to obtain a high level of evidence for clinical application. Fourth, more efficient image preprocessing tools (80) need to be incorporated to improve the robustness and versatility of the method.

## Conclusion

In conclusion, using a combination of clinical features and radiomics, we developed and validated a set of methods for the early prediction of stroke prognoses. We combined DNN ideas with ensemble learning to use OEDL as an effective tool for the early and non-invasive prediction of prognosis levels, thereby optimizing the clinical decision-making process and improving the efficiency of clinical intervention. The ideas in this study can provide new research directions for the effective establishment of stroke prevention and control mechanisms.

## Data availability statement

The raw data supporting the conclusions of this article will be made available by the authors, without undue reservation.

## Ethics statement

Ethical review and approval was not required for the study on human participants in accordance with the local legislation and institutional requirements. Written informed consent from the patients/participants or patients/participants' legal guardian/next of kin was not required to participate in this study in accordance with the national legislation and the institutional requirements.

## Author contributions

WY: conceptualization, data curation, resources, methodology, software, formal analysis, validation, investigation, and writing—original draft. XC: methodology, software, formal analysis, validation, investigation, writing—original draft, and editing and polishing. PL: data curation, resources, software, and formal analysis. YT: software, validation, investigation, and data curation. ZWa: validation, investigation, data curation, and editing and polishing. CG: investigation, resources, and writing—polishing. JC: validation and resources. FL: formal analysis and validation. DaY and ZWe: writing—polishing. DoY: conceptualization, investigation, validation, and resources. YW: funding acquisition, conceptualization, investigation, resources, methodology, writing—review and editing, project administration, and supervision. All authors contributed to the article and approved the submitted version.
